# Aspirin in diabetic patients at primary prevention: insights of the VITAL cohort

**DOI:** 10.1007/s40618-022-02001-3

**Published:** 2023-01-18

**Authors:** D. Caldeira, M. Alves, J. J. Ferreira, F. J. Pinto

**Affiliations:** 1 Centro Cardiovascular da Universidade de Lisboa–CCUL (CCUL@RISE), Faculdade de Medicina, CEMBE, CAML, Universidade de Lisboa, Lisbon, Portugal; 2 Serviço de Cardiologia, Hospital Universitário de Santa Maria–CHULN, Lisbon, Portugal; 3 Laboratory of Clinical Pharmacology and Therapeutics, Faculdade de Medicina, Universidade de Lisboa, Lisbon, Portugal; 4 Faculdade de Medicina, Instituto de Medicina Molecular, Universidade de Lisboa, Lisbon, Portugal; 5 Serviço de Medicina III, Hospital Pulido Valente, CHLN, Lisbon, Portugal

**Keywords:** Cardiovascular disease, Primary prevention, Aspirin, Diabetes

## Abstract

**Purpose:**

Aspirin use among patients with diabetes in primary prevention is still a matter of debate. We aimed to evaluate the potential cardiovascular risk benefit of aspirin in primary prevention, using data from a contemporary cohort.

**Methods:**

Retrospective analysis of the VITAL cohort with > 20,000 individuals at primary prevention who were followed for a median of 5.3 years. The population was evaluated according to the baseline diabetes status, and then aspirin use was evaluated among diabetic patients. Cox regression models were used to estimate the risks of mortality and cardiovascular outcomes. The estimates were reported using adjusted hazard ratio (HR) and 95% confidence intervals (95%CI).

**Results:**

Diabetic patients (*n* = 3549; 13.7%) showed to increase the risk of all-cause mortality (HR 1.61, 95%CI 1.33–1.94), and major adverse cardiovascular events (MACE) (HR 1.36 95%CI 1.11–1.68) than non-diabetic population. Diabetic patients taking aspirin were older, more frequently man, hypertensive, current users of statins, and current smokers compared with diabetic patients who did not use aspirin at baseline. There was no difference between diabetic aspirin users and non-users regarding all-cause mortality (HR 0.80, 95%CI 0.59, 1.10), MACE (HR 0.92, 95%CI 0.64, 1.33), coronary heart disease (HR 0.98, 95%CI 0.67, 1.43), or stroke (HR 0.87, 95%CI 0.48, 1.58).

**Conclusions:**

The VITAL data confirmed diabetes as an important risk factor for cardiovascular events in a contemporary cohort but did not show cardiovascular benefits of aspirin in primary prevention among people with diabetes who were shown to be at higher risk of cardiovascular events.

**Supplementary Information:**

The online version contains supplementary material available at 10.1007/s40618-022-02001-3.

## Introduction

Diabetes is a well-known risk factor for major cardiovascular disease (CVD) and is deemed to increase at least twice the risk of atherosclerotic disease compared with non-diabetic patients [1–4].

Aspirin is commonly used in the treatment and prevention of CVD, and the effectiveness of aspirin for the secondary prevention of CVD is well established in people with or without diabetes. In contrast, the role of aspirin in primary prevention is still controversial, and conceptually an overall benefit can be achieved if the baseline risk is high enough to outweigh the risks of aspirin side effects. Diabetic patients were deemed to fulfill these criteria but dedicated randomized controlled trials failed to show a clear net benefit of aspirin for the primary prevention of CVD in people with diabetes [5].

Nevertheless, guidelines still suggest that aspirin might be considered for the primary prevention in selected patients at higher CVD risk but not at increased bleeding [6].

In order to further explore the risk of CVD risk among diabetic patients and the putative benefit hypothesis of aspirin in this population at primary prevention we conducted a retrospective analysis of data from VITAL (Vitamin D and Omega-3 Trial) cohort, majorly composed by patients with less than 70 years [7].

## Subjects, materials, and methods

This was a retrospective evaluation of the VITAL trial using data provided by the Project Data Sphere (https://doi.org/10.34949/n4c7-zm25). Detailed methodology and main results of the trial has been published elsewhere [8]. In summary, 25 828 adults (men age ≥ 50 and women age ≥ 55 years) were followed for a median of 5.3 years (range, 3.8–6.1 years), since July 2010. VITAL was a 2 × 2 factorial double-dummy randomized controlled trial (RCT) evaluating Vitamin D and/or omega-3 fatty acids vs placebo for primary prevention of cancer and cardiovascular disease. The dataset used is de-identified and public according to request to PDS. This project complies with the principles of the Declaration of Helsinki as revised in 2008.

### Participants

The VITAL trial was performed in the United States in patients at primary prevention. Therefore, participants were required to have no history of cancer (except non-melanoma skin cancer), myocardial infarction, stroke, transient ischemic attack (TIA), angina pectoris, or coronary revascularization. In addition, participants were required to limit consumption of supplemental vitamin D, to limit consumption of supplemental calcium, and to forego the use of fish oil supplements during the run-in and randomized treatment periods. Patients with renal failure or dialysis, hypercalcemia, hypo- or hyperparathyroidism, severe liver disease (cirrhosis), or sarcoidosis or other granulomatous diseases, such as active chronic tuberculosis or Wegener's granulomatosis, were excluded [8].

For our retrospective analysis participants were split according to the diagnosis of diabetes at admission and aspirin use.

### Outcomes

The primary method of follow-up was by mailed questionnaires and reviewing medical records to confirm study endpoints. Participants received follow-up questionnaires at 6 months and 1 year after randomization and annually thereafter. An Endpoints Committee of physicians who were blinded to the randomized treatment assignment reviewed the medical records to confirm or refute the case by applying a defined protocol [8].

The primary cardiovascular outcome of the VITAL study was major cardiovascular adverse events (MACE), a composite endpoint of myocardial infarction, stroke, and death from cardiovascular causes. The definition of Myocardial infarction (MI) was that of Joint European Society of Cardiology/American College of Cardiology Foundation/American Heart Association/World Heart Federation Task Force for the Redefinition of Myocardial Infarction criteria. Stroke was diagnosed and categorized according to Trial of Org 10,172 in Acute Stroke Treatment (TOAST) criteria. Cardiovascular deaths were confirmed by convincing evidence of a CVD event from all available sources, including death certificates, hospital records, autopsy reports, and, for deaths outside the hospital, observer accounts. Total coronary heart disease (CHD) was a composite of myocardial infarction, coronary revascularization (percutaneous coronary intervention or coronary artery bypass grafting), and death from coronary heart disease. Expanded MACE was a composite of MACE and coronary revascularization (coronary artery bypass grafting or percutaneous coronary intervention) [8, 9].

### Statistical methods

Participants of the cohort were split according with the baseline diabetes status and in a second phase the diabetic participants were split according to aspirin usage. Descriptive analysis was performed, mean and standard deviation were presented for continuous variables and absolute and relative frequencies for categorical variables. Statistical comparisons were performed using t-test for independent variables to test for continuous values and Chi-square test for categorical variables. Cox regression models were applied to assess the risk estimates for univariate models and multivariate models. The tied events were handled through the Breslow method, and the Schoenfeld residuals were used to test the assumption of proportional hazards method. Hazard Ratio (HR) and 95%CI were estimated. A significance level of 5% was assumed for all statistical calculations. Analyses were performed using the STATA statistical software program (STATA version 17.0; StataCorp, College Station, TX).

## Results

### Diabetes risk for cardiovascular events in the VITAL cohort

**Fig. 1:**
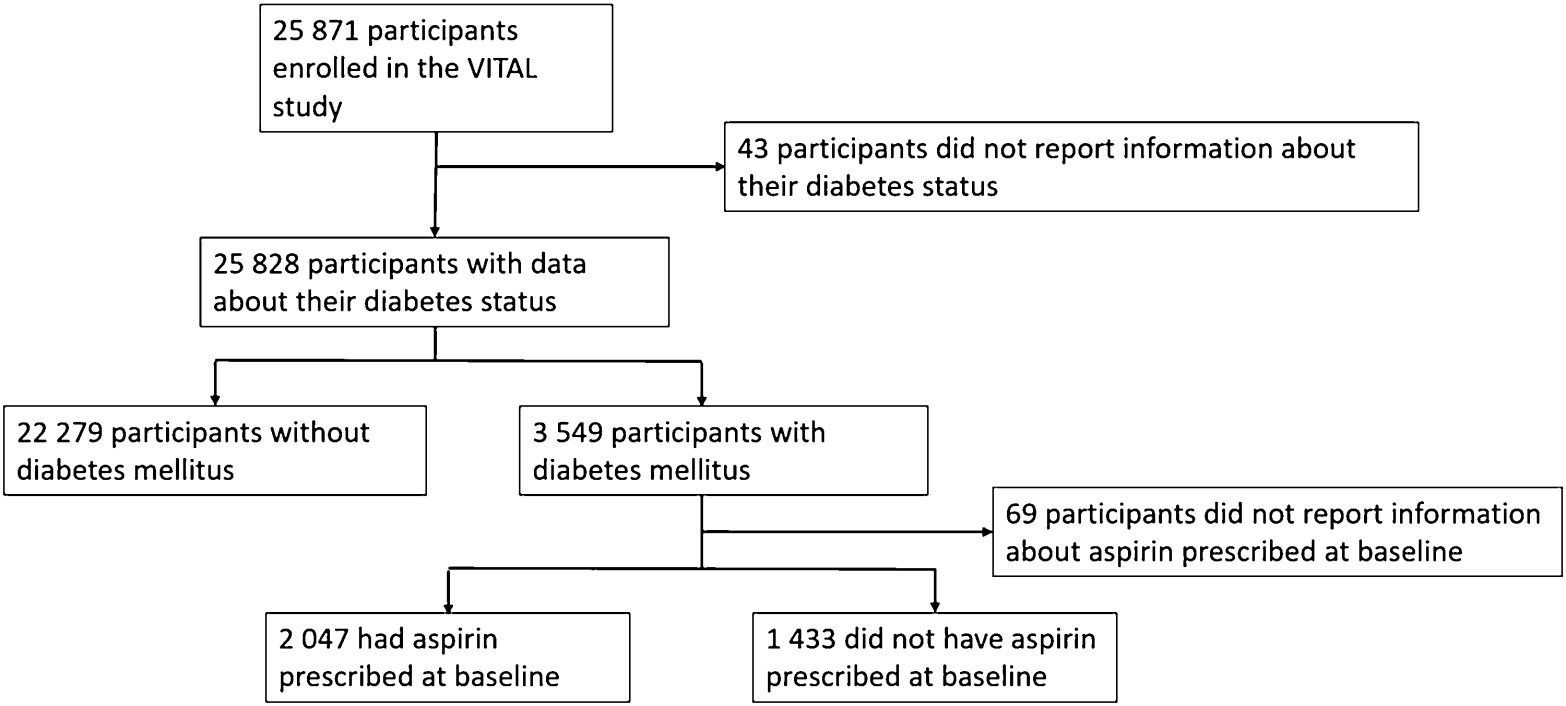
Flowchart showing the patients according to baseline diabetes status and aspirin use in diabetics

Among 25,828 participants with information about the baseline diabetes status, 13.7% (*n* = 3549) were diabetic (Fig. 1). The mean age of the participants was 66.6 years and there were no significant differences regarding age in diabetics and non-diabetic participants, nor in the proportion of male/female participants. Diabetic patients had a higher prevalence of other cardiovascular risk factors, such as obesity, hypertension, dyslipidemia, current smoking, and parental history of MI (Table 1). The frequency of aspirin use was higher among patients with diabetes compared with non-diabetics (58.8% vs. 43.2%) in this primary prevention population (Table 1).

**Table 1:** Baseline characteristics

	Global VITAL cohort (*n* = 25,828)		*P* value	Diabetic participants (*n* = 3549)		*P* value
	Diabetes (*n* = 3549)	No Diabetes (*n* = 22,279)		Aspirin (*n* = 2047)	No aspirin (*n* = 1433)	
Age (years) (mean, SD)	66.6 (7.0)	66.6 (7.1)	0.720	67.0 (6.8)	66.0 (7.2)	< 0.001
Age > 70 years (%, *n*)	30.4 (1079)	30.3 (6760)	0.942	31.9 (654)	28.1 (402)	0.014
Sex (men) (%, n)	48.4 (1718)	49.6 (11,043)	0.200	51.3 (1051)	44.6 (639)	< 0.001
BMI (kg/m2) (mean, SD)	31.9 (6.9)	27.5 (5.3)	< 0.001	31.9 (6.8)	31.9 (7.0)	0.908
Obese (%, *n*)	52.6 (1867)	24.3 (5405)	< 0.001	52.5 (1074)	53.2 (762)	0.680
Hypertension (%, *n*)	79.7 (2799)	45.1 (9976)	< 0.001	82.6 (1676)	75.3 (1065)	< 0.001
Baseline statins (%, *n*)	60.4 (2092)	30.9 (6788)	< 0.001	67.1 (1361)	50.5 (714)	< 0.001
Current smoking (%, *n*)	9.2 (321)	6.9 (1511)	< 0.001	7.6 (154)	11.6 (165)	< 0.001
Parental history of MI (%, *n*)	19.2 (585)	15.5 (3064)	< 0.001	20.2 (360)	17.7 (215)	0.09
Aspirin use (%, *n*)	58.8 (2047)	43.2 (9504)	< 0.001	100 (2047)	0 (0)	–

*BMI* Body mass index, *MI* Myocardial infarction, *SD* Standard deviation

**Fig. 2:**
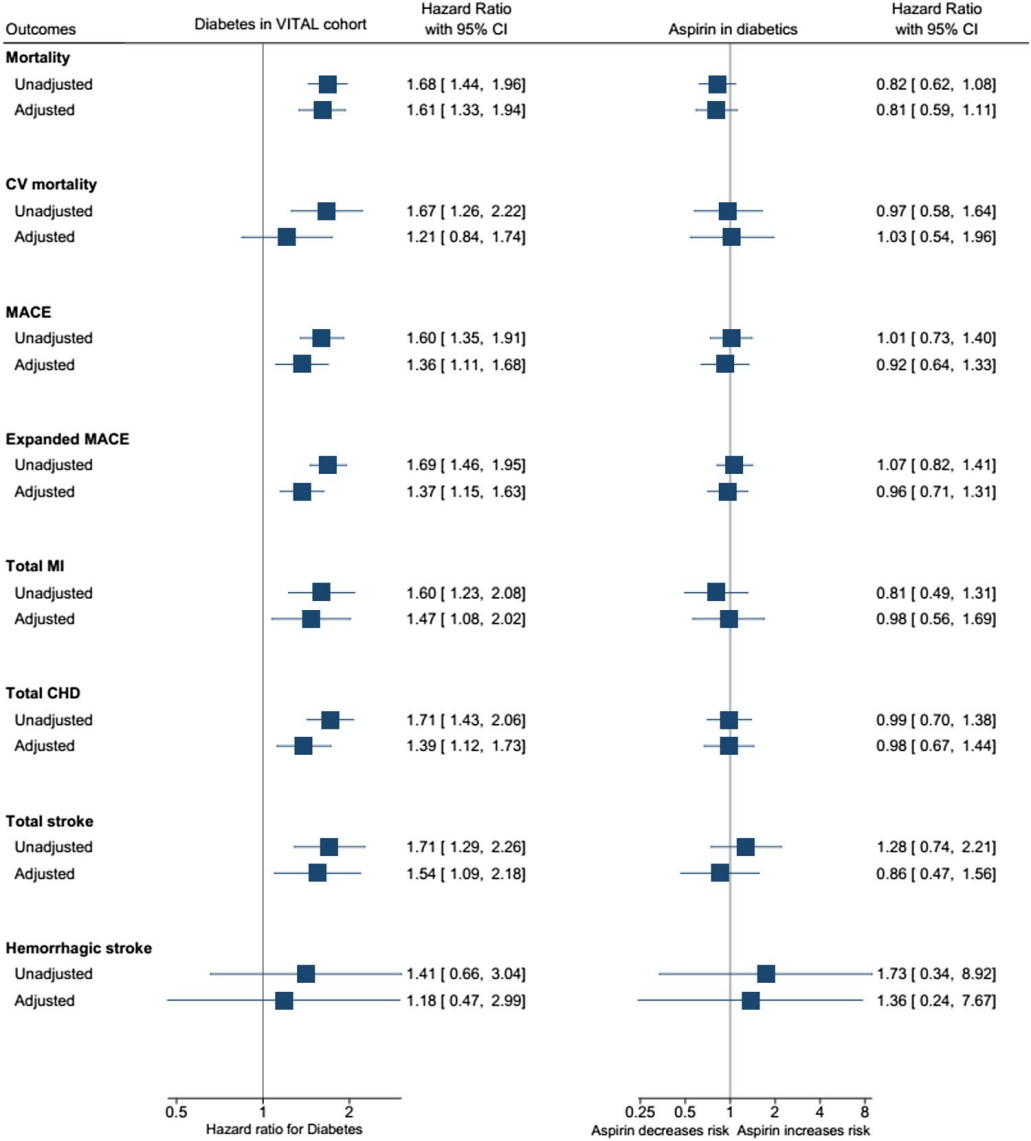
Plot showing the estimates (unadjusted and adjusted) in hazard ratios for diabetes (left side) and for aspirin in diabetic patients (right side). Adjusted estimates accounted for age, sex, body mass index, smoking status, parental history of myocardial infarction, hypertension, statins use. CHD: Coronary heart disease; *CI* Confidence interval, *CV* Cardiovascular, *MACE* Major adverse cardiovascular events, *MI* Myocardia infarction

**Fig. 3:**
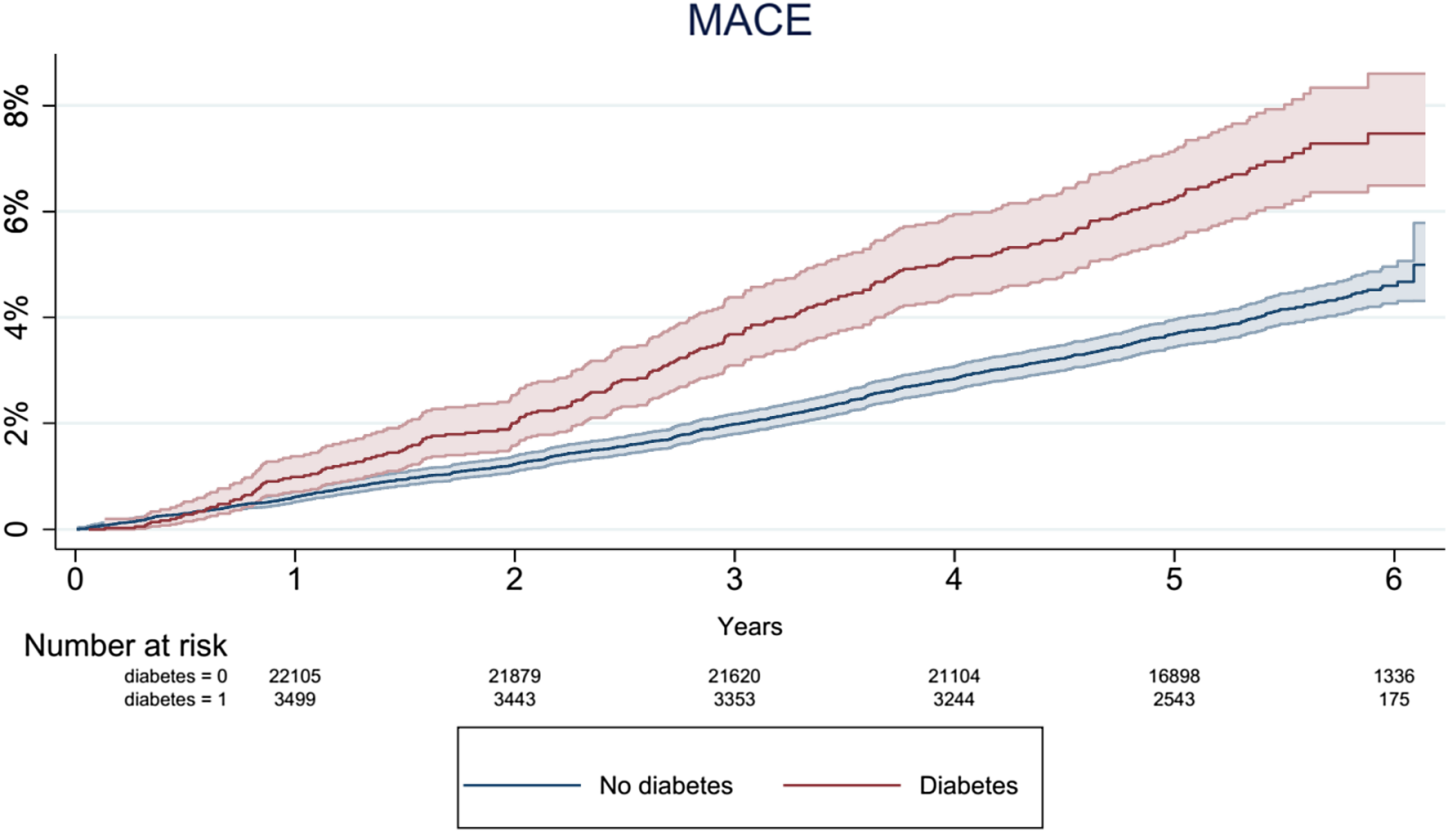
Cumulative hazard plot showing the increased risk of Major Adverse Cardiovascular Events (MACE) of diabetic patients compared to non-diabetic individuals

After adjusting for potential confounder, people with diabetes had increased risk of mortality (HR 1.61, 95%CI 1.33–1.94) and higher risk of cardiovascular outcomes, such as MACE (HR 1.36 95%CI 1.11–1.68), expanded MACE (HR 1.37, 95%CI 1.15–1.63), MI (HR 1.47, 95%CI 1.08–2.02), coronary heart disease (HR 1.39, 95%CI 1.12–1.73), and stroke (HR 1.54, 95%CI 1.09–2.18), when compared to non-diabetic population (Fig. 2). Figure 3 shows the cumulative hazard plot of MACE comparing diabetic and non-diabetic individuals. Figure 2 shows in the left side of the plot the unadjusted and adjusted estimates associated with diabetes compared to non-diabetic participants. Supplementary Table 1 shows the number of events and the incidence rate among the groups.

### Association of baseline aspirin use in diabetic patients with cardiovascular events

Among participants with diabetes, aspirin users were older (67 vs 66 years old) and more frequently men (51 vs 45%, *p* < 0.001). There were no differences regarding obesity between the groups. At baseline 82.6% of aspirin users had hypertension, significantly higher compared to non-users (vs. 75.3%, *p* < 0.001). The proportion of statins users and current smokers was also higher in diabetics treated with aspirin (Table 1).

After adjusting for age, sex, body mass index, smoking status, parental history of myocardial infarction, hypertension, and statins use, aspirin was not associated with decreased risk of cardiovascular events (MACE: HR 0.92, 95%CI 0.64–1.33) nor all-cause mortality (HR 0.81, 95%CI 0.59–1.11) (Fig. 2). Figure 2 shows in the right side of the plot the unadjusted and adjusted estimates associated with aspirin use diabetic patients. Supplementary Table 1 shows the number of events and the incidence rate among the groups.

## Discussion

In this post hoc retrospective analysis of the VITAL study, we confirmed that diabetes had a significant impact in the mortality and cardiovascular outcomes among individuals at primary prevention and that aspirin use in diabetic patients in this setting was not associated with risk reduction of mortality or cardiovascular events.

It is important to stress that patients treated with aspirin had high frequency of cardiovascular risk factors, such as older age, male gender, hypertension, and smoking habits. Data from diabetes vs no diabetes are concordant with literature and support the increased risk of cardiovascular events associated with diabetes [1], and at least partially explain the willingness to prescribe aspirin in these patients [10]. However, the magnitude of risk increase with diabetes was not high as previously reported [4], which can be related to the standard practices for care of cardiovascular risk factors. And despite the adjustments of estimates for different potential cofounder the putative expected benefit of aspirin in diabetic patients was not documented in this cohort.

Previous systematic review of RCTs, including more than 27,000 diabetic patients, suggested that aspirin use among people with diabetes increased risk of major bleeding and major gastrointestinal bleeding with a modest 8% risk reduction of MACE and no mortality benefit [11]. Additionally, the small benefit of aspirin mostly relied on older and small clinical trials, while recent trials, such as ASCEND, showed that the risk–benefit of aspirin in contemporary population might be weak [12]. Oppositely to the data from RCT, in observational studies the groups might not balanced in terms of cardiovascular risk factors and comparison are at higher risk of bias also due to possible indications bias related to aspirin prescription (i.e., aspirin is prescribed preferentially in individuals at higher cardiovascular risk). In our analyses we attempted to adjust for these potential sources of bias, but we acknowledge that residual bias can affect the estimation of the results.

After many years promoting aspirin benefits among patients, deprescribing aspirin in primary prevention in patients with low bleeding risk could be challenging to physicians. Although data regarding cardiovascular events after deprescribing aspirin in low/moderate CVD risk patients is lacking, previous Swedish study stated that discontinuing primary prevention aspirin in general population was associated with a 28% higher rate of cardiovascular events than continuing on aspirin, which result in an absolute risk increase of 6.9 per 1000 person-years at risk or an additional cardiovascular event per year in 1 of every 146 patients who discontinued aspirin [13]. However, no data about bleeding complications risk prevented by deprescription were supplied.

Our retrospective analysis of the VITAL cohort has some limitation that should be acknowledged. First it is important to notice that VITAL participants were health-conscious group of people, keen to participate in a clinical trial with the intention to reduce their CVD or cancer risk.  [14] So, data could not be generalized to less health-conscious people and with possibly very high CVD risk. Another limitation of our study is the retrospective nature of our analysis and the possible residual bias despite the adjustments made in the analyses. The lack of comprehensive data regarding overall major bleeding risk, in particular gastrointestinal bleeding, could also be a pitfall.

## Conclusions

This retrospective analysis of the VITAL study showed that diabetes significantly increases the risk of cardiovascular events in a contemporary cohort of patients at primary prevention. No association was found between aspirin and cardiovascular benefits of aspirin in these diabetic patients at primary prevention.

## Supplementary Information

Below is the link to the electronic supplementary material.

Supplementary file1 (DOCX 18 KB)

## Data Availability

We used the data provided by the Project Data Sphere (10.34949/n4c7-zm25). Detailed methodology and main results of the trial have been published elsewhere. The dataset used is de-identified and public according to request to PDS.
