# Supplementary material for: Aspirin in diabetic patients at primary prevention: insights of the VITAL cohort
Source: J Endocrinol Invest. 2023 Jan 18;46(7):1423–8. doi: 10.1007/s40618-022-02001-3 (PMC10261209; doi:10.1007/s40618-022-02001-3)
Supplement: Supplementary file 1 — Supplementary file1 (DOCX 18 KB) [file 40618_2022_2001_MOESM1_ESM.docx]

Supplementary Table 1: Events and incidence rates among diabetic and non-diabetic individuals of VITAL cohort.

| Outcomes | Events  % (n) | | Incidence per 1000-PY  (95%CI) | |
| --- | --- | --- | --- | --- |
|  | **Diabetes**  **(n=3549)** | **No diabetes**  **(n=22279)** | **Diabetes** | **No diabetes** |
| All-cause Mortality | 5.7%  (204) | 3.8%  (773) | 11.0  (9.6-12.6) | 6.6  (6.1-7.1) |
| CV mortality | 1.7%  (60) | 1.0%  (229) | 3.2  (2.5-4.2) | 1.9  (1.7-2.2) |
| MACE | 4.5%  (161) | 2.9%  (642) | 8.8  (7.6-10.3) | 5.5  (5.1-6.0) |
| Expanded MACE | 6.4%  (227) | 3.9%  (865) | 12.6  (11.0-14.3) | 7.5  (7.0-8.0) |
| Total MI | 1.9%  (69) | 1.2%  (275) | 3.8  (2.9-4.7) | 2.4  (2.1-2.6) |
| Total CHD | 4.0%  (143) | 2.4%  (534) | 7.9  (6.7-9.3) | 4.6  (4.2-5.0) |
| Total Stroke | 1.7%  (61) | 1.0%  (228) | 3.3  (2.6-4.3) | 1.9  (1.7-2.2) |
| Hemorrhagic stroke | 0.2%  (8) | 0.2%  (36) | 0.4  (0.2-0.9) | 0.3  (0.2-0.4) |

CHD: Coronary heart disease; CI: Confidence interval; CV: Cardiovascular; MACE: Major adverse cardiovascular events; MI: Myocardia infarction; PY: patient-years

Supplementary Table 2: Events and incidence rates among aspirin and non-aspirin users among diabetic patients.

| Outcomes | Events in diabetics  % (n) | | Incidence per 1000-PY in diabetics  (95%CI) | |
| --- | --- | --- | --- | --- |
|  | **Aspirin**  **(n=2047)** | **No aspirin**  **(n=1433)** | **Aspirin** | **No aspirin** |
| All-cause Mortality | 109  (5.3%) | 91  (6.4%) | 10.1  (8.4-12.2) | 12.2  (10.0-15.1) |
| Cardiovascular mortality | 34  (1.7%) | 24  (1.7%) | 3.2  (2.3-4.4) | 3.2  (2.2-4.8) |
| MACE | 91  (4.4%) | 62  (4.3%) | 8.6  (7.0-10.6) | 8.5  (6.6-10.9) |
| Expanded MACE | 133  (6.5%) | 86  (6.0%) | 12.6  (10.7-15.1) | 11.9  (9.6-14.6) |
| Total MI | 35  (1.7%) | 30  (2.1%) | 3.3  (2.4-4.6) | 4.1  (2.8-5.8) |
| Total CHD | 81  (4.0%) | 57  (4.0%) | 7.7  (6.2-9.5) | 7.8  (6.0-10.1) |
| Total Stroke | 37  (1.8%) | 20  (1.4%) | 3.5  (2.5-4.8) | 2.7  (1.8-4.2) |
| Hemorrhagic stroke | 5  (0.2%) | 2  (0.1%) | 0.5  (0.2-1.1) | 0.3  (0.1-1.1) |

CHD: Coronary heart disease; CI: Confidence interval; CV: Cardiovascular; MACE: Major adverse cardiovascular events; MI: Myocardia infarction; PY: patient-years.
